# Supporting the transition to higher education: examination of state- and trait-level effects of a multicomponent positive psychological intervention for university students

**DOI:** 10.3389/fpsyg.2025.1631713

**Published:** 2025-08-12

**Authors:** Christina Ewert, Lou Palme, Wilhelm H. A. Voigt, Samuel Tomczyk

**Affiliations:** ^1^Department of Psychology, University of Potsdam, Potsdam, Germany; ^2^Institute of Psychology, University of Greifswald, Greifswald, Germany

**Keywords:** positive psychology, intervention, state-trait, university students, Ecological momentary assessment

## Abstract

**Introduction:**

Positive psychological interventions (PPIs) can help mitigate psychological challenges and facilitate the transition to higher education.

**Methods:**

This study presents the results of a quasi-experimental trial of a 6-week universal, multicomponent PPI designed specifically for university students. It compares survey responses of intervention group participants with those of passive control group participants before, immediately after, and 6 months after the intervention.

**Results:**

The results show that the PPI led to significant improvements in mindfulness, positive reframing, and self-compassion at the trait level, and in self-compassion, positive affect, relaxation, subjective physical health, and subjective sleep quality at the state level.

**Discussion:**

The study demonstrates that PPIs can effectively build psychological resources during educational transitions, particularly those related to emotion regulation. However, other resources and outcomes (e.g., character strengths) were less affected. Thus, more tailored approaches and selective interventions are recommended to address these areas comprehensively and ensure a more holistic improvement in student wellbeing.

## 1 Introduction

Transitioning from secondary to post-secondary education is an exciting yet challenging time for early adults, characterized by various changes (Ketchen Lipson et al., 2015). While such upheavals can unlock potential for positive psychosocial development, such as forming close relationships, they simultaneously increase stress levels and, as a result, vulnerability to developing mental illness (Erikson, 1959; Kroshus et al., 2021). Additional external stressors like the coronavirus pandemic have affected university students. Studies suggest that stress, depression, loneliness, and anxiety have increased since the start of the coronavirus pandemic (Aristovnik et al., 2020; Kohls et al., 2023; Tsiouris et al., 2023). The rise in mental impairment coincides with increased workload, isolation, and financial burdens (Becker and Lörz, 2020; Kreidl and Dittler, 2021; Lörz et al., 2021). Even post-pandemic, students’ mental health remains impaired compared to pre-pandemic levels, with a reduction in social support, resilience, and self-efficacy (Kohls et al., 2023). Hence, there is an urgent need for interventions addressing students’ mental health.

To this end, positive psychology offers a promising approach by emphasizing the development of positive resources and skills, focusing on enhancing wellbeing rather than just treating pathology (Seligman and Csikszentmihalyi, 2000). Research indicates that PPIs implemented in university settings promote mental health of first-year students and facilitate the transition to university (Dvořáková et al., 2017; Hirshberg et al., 2022). Participating in a PPI in a university context can lead to a reduction in perceived stress, depression, negative affect, and anxiety and an increase in wellbeing, life satisfaction, mindfulness, self-compassion, optimism and self-efficacy (Dvořáková et al., 2017; Hirshberg et al., 2018; Hirshberg et al., 2022; Hobbs et al., 2022; Lorenz et al., 2022; Smeets et al., 2014; Totzeck et al., 2020). Various types of PPIs exist, some focusing only on single components, such as gratitude (Emmons and McCullough, 2003), and others incorporating multiple components of positive psychology (Chilver and Gatt, 2022). A meta-analysis by Hendriks et al. (2020) reported a significant impact of multicomponent PPIs on increasing subjective wellbeing (Hedges’ *g* = 0.34), psychological wellbeing (Hedges’ *g* = 0.39), and reducing depression (Hedges’ *g* = 0.29), as well as anxiety and stress (Hedges’ *g* = 0.35).

Psychological constructs can be conceptualized at both the trait level, representing relatively stable, enduring characteristics, and the state level, reflecting transient psychological states that fluctuate across situations (Schmitt and Blum, 2020). Traditionally, research on positive psychological interventions (PPIs) has focused primarily on trait-level changes, assuming that wellbeing-related constructs develop gradually over time (Hendriks et al., 2020). However, constructs such as mindfulness, self-compassion, and affect can also be examined at the state level, providing insights into short-term, dynamic changes that occur in response to interventions (Moskowitz et al., 1997; Blanke and Brose, 2017). According to Whole Trait Theory, traits emerge from accumulated state experiences, meaning that repeated state-level improvements may contribute to long-term trait development (Schmitt and Blum, 2020). Traits, thus, represent a density distribution of states which may very within persons and across situations. Whole Trait Theory provides a framework to examine interpersonal (e.g., gender) and intrapersonal differences in states as well as social-cognitive predictors of variety (e.g., attitudes, knowledge). For intervention research, this is particularly important as it allows us to study intervention effects on inter- and intra-personal levels. Yet, not all constructs are equally responsive to interventions at both levels—some (e.g., mindfulness and self-compassion) may show immediate state improvements, whereas others (e.g., character strengths) may require extended practice for trait-level change (Kiken et al., 2015; Neff and Germer, 2013). By differentiating between state and trait outcomes, this study aims to clarify how PPIs influence psychological resources over different timescales and deepen our understanding of their mechanisms of action.

Despite ample evidence of PPI effectiveness in university students, several questions remain unanswered. First, multicomponent PPIs are underutilized compared to single-component interventions (Schotanus-Dijkstra et al., 2015), despite evidence suggesting their superior effectiveness (Schueller and Parks, 2012). For instance, a meta-analysis by Carr et al. (2021) showed that multicomponent PPIs were more effective than single-component interventions in reducing depression and stress as well as in increasing wellbeing. Second, most PPIs do not differentiate between trait and state levels in their intervention or analysis but examine trait levels alone. Consequently, they view the trait level as general, stable, situation-independent characteristics (Schmitt and Blum, 2020). However, constructs can also be viewed at the state level as patterns of thinking, feeling and behavior of a person in a particular situation that can fluctuate over time (Schmitt and Blum, 2020). It is more plausible that all constructs comprise both state and trait components, making a strict separation between the two inherently difficult (Moskowitz et al., 1997). Consequently, immediately after the intervention, there may be a more pronounced increase at state level compared to trait level of the constructs. In that sense, long-term follow-ups can provide further insight into the development of state- and trait-level variables. Lastly, multicomponent PPIs are usually delivered by mental health professionals or apps. Yet the potential of peer-to-peer approaches, which may bring benefits to effectiveness and cost-efficiency remains untapped (Neuhaus et al., 2022).

To address these questions, this study examines the effects of a 6-week PPI on German university students focusing on both state- and trait-level changes in positive psychological outcomes. The intervention is based on the Positive Psychotherapy approach by Rashid and Seligman (2021) and was co-created with university students via focus group work (*n* = 8) conducted over 6 months, followed by a pilot trial across two semesters (*n* = 40). The Positive Psychotherapy approach was adapted to the most common mental health problems among students (i.e., symptoms of depression and anxiety) and the number of sessions was reduced to six, since this was found to be feasible in the pilot study. The PPI addresses six primary positive psychological outcomes, namely character strengths, mindfulness, positive reframing, self-compassion, humor, and gratitude. These were identified as efficacious in previous research and meaningful by the focus group and in the pilot study (Carr et al., 2021). Moreover, in designing the course, all constructs were mapped on the PERMA model (Seligman, 2011) and connected to didactic approaches to ensure that positive emotions (e.g., gratitude), engagement (e.g., use of character and signature strengths), relationships (e.g., humor as a tool for social connectedness), meaning (e.g., using mindfulness and self-compassion to identify values), and accomplishment (e.g., using positive reframing to increase self-efficacy) were addressed. As secondary outcomes, we measure various constructs such as stress, wellbeing, depression, and others, based on previous research (Carr et al., 2021; Hendriks et al., 2020). We predict that participants in the intervention group show significant improvements in primary and secondary outcomes from pretest to posttest as well as follow-up (6 months after the intervention). Moreover, we explore the impact of the intervention at the state-level by examining daily diaries of outcomes before and after the intervention.

## 2 Materials and methods

### 2.1 Procedure

We recruited participants through flyers, social media (e.g., Instagram), and university channels (university’s sports department and the student health management website).

**Figure 1:**
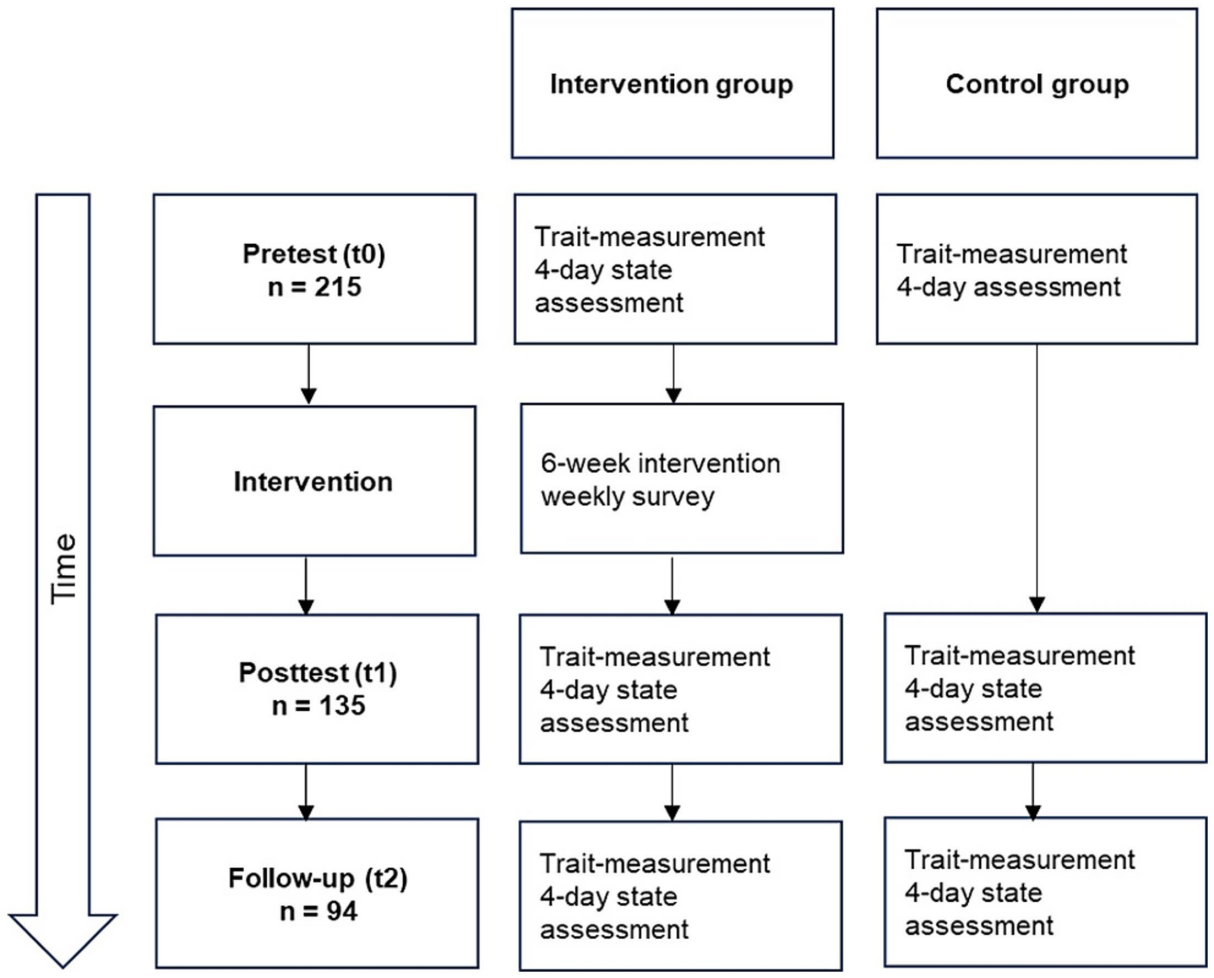
Study procedure. Intervention group and control group complete trait- and state-assessments before, after and 6 months after the intervention. The number of participants decreases from 215 to 135 after the intervention and down to 94 at follow-up.

We recruited the passive control group using the university’s internal pool of test subjects. There was no randomized allocation of participants, because the program was part of the universities’ health initiative. Participants primarily enrolled with the motivation to receive an intervention and might have dropped out if they had been assigned to the control group. Additionally, we advertised among private friends and acquaintances and published university board notices. Study-registration was implemented via the university’s sports department and the student health management website. We contacted the participants via e-mail, which informed them about the procedure and conditions of participation. Following this, they provided their informed consent. The subsequent study procedure is presented in Figure 1.

One week prior to the beginning of the intervention, the participants completed a trait assessment via SoSci Survey^1^ and engaged in a 4-day state assessment (i.e., daily diaries). The trait assessment consisted of a 30-min questionnaire that covered different trait constructs. The state assessment consisted of a daily 5-min evening survey conducted over 4 consecutive days via SoSci Survey. After the pretest phase, the intervention group participants took part in the 6-week positive psychological intervention. The intervention consisted of six modules, with one 90-min module per week. The passive control group did not receive any intervention. After the intervention phase, participants completed the trait and state assessments again. Six months after the intervention, we conducted the follow-up assessment, during which the participants once again completed the state and trait surveys.

Data collection started in October 2022 (t0), followed by the 6-week intervention and a posttest in December 2022 (t1). Follow-up data was collected in summer 2023 (t2). To achieve the estimated sample size, we implemented a similar assessment schedule with another wave of participants for the summer semester 2023 and used data of both waves for the analysis.

### 2.2 Participants

A total of 215 students from two German universities enrolled in the study (intervention group *n* = 131, passive control group *n* = 84). Inclusion criteria were speaking and understanding German, and being enrolled in university. People were excluded if they were currently receiving psychotherapy and their therapist advised against participating. Demographic data was available for 212 participants. The participants were 18–42 years old, with an average age of 22.93 years (SD = 4.48). The participants were 79.1% female (*n* = 170), 16.3% male (*n* = 35), 2.3% diverse (*n* = 5) and 0.9% did not provide any information about their sex/gender (*n* = 2; three missing values for age). Most students were in their first or third semester (*n* = 132, 61.4%), 8.8% (*n* = 19) were in their second semester, 28.37% (*n* = 61) were in their fourth semester and higher. Most students were studying for a Bachelor’s degree (Bachelor of Science: *n* = 152, 70.7%; Bachelor of Arts: *n* = 16, 7.4%), 7.4% were studying for a state examination (*n* = 16), 6% were studying for a Master’s degree (Master of Science: *n* = 11, 5.1%; Master of Arts: *n* = 2, 0.9%), 0.9% were studying for a diploma (*n* = 2) and 6% were studying for another degree (*n* = 13).

### 2.3 Intervention

The intervention consisted of 6 weeks of training in various positive psychological skills. The program was delivered as peer education by students from two German universities supported by two faculty members. The intervention followed a peer-to-peer approach, with students not only co-developing but also delivering the program to participants (Byl et al., 2016; Schneider et al., 2024). The student facilitators were paired with another person from the partner university to co-develop and standardize the delivery method of the workshop. All facilitators participated in a 2-day training period where each workshop was practiced and a standard was agreed upon for each. Since the program was co-designed with participants, it considered the university context, organizational aspects, and culture of the target group from the beginning (Topping, 2022). The training aimed to support students in coping with the demands and challenges of studying in a healthy way in the initial phase of their studies. A total of six modules of 90 min each were offered, with each module forming a separate thematic block. The following topics were covered: character and signature strengths, gratitude, mindfulness, positive reframing, self-compassion and humor. Each module included a theoretical introduction, practical exercises, and tips for everyday transfer (a detailed description of each module is presented in the OpenScienceFramework project). After the sessions, the participants received worksheets and brief tasks for home practice.

### 2.4 Measures

The survey consisted of several validated scales and some original items to measure sociodemographic covariates, positive psychological skills as states and/or traits, and health-related outcomes. Primary outcomes were the constructs addressed in the intervention, while secondary outcomes includes other (mental) health related and positive psychological constructs. State measures were used for constructs where daily fluctuation may occur. An overview of measures is presented in Table 1.

**Table 1:** Trait and state measures.

Construct (*n* items)	Measure		*α*			Scale
Traits			T0	T1	T2	
Character Strengths (5)	Subscale of the Positive Psychotherapy Inventory, German version (Rashid and Seligman, 2021)		0.74	0.8	0.82	1 (*not true at all*) to 5 (*completely true*)
Mindfulness (14)	Freiburger Fragebogen zur Achtsamkeit Short-Form (Walach et al., 2004)		0.86	0.89	0.88	1 (*almost never*) to 4 (*almost always*)
Positive Reframing/ Reappraisal (6)	Subscale of the Emotion Regulation Questionnaire, German version (Abler and Kessler, 2009)		0.84	0.86	0.88	1 (*not true at all*) to 7 (*completely true*)
Self-Compassion (26)	Self-Compassion Scale Long-Form, German version (Hupfeld and Ruffieux, 2011)		0.92	0.94	0.94	1 (*almost never*) to 5 (*almost always*)
Coping Humor (7)	Coping-Humor Scale, German version (Leicht, 2015; Martin and Lefcourt, 1983)		0.73	0.79	0.79	1 (*strongly disagree*) to 4 (*strongly agree*)
Wellbeing (5)	WHO-5 (World Health Organization, 1998)		0.86	0.86	0.85	0 (*at no time*) to 5 (*all the time*)
Wellness Behavior (12)	Wellness Behavior Inventory, German version (Sirois, 2001)		0.68	0.61	0.62	1 (*less than once a week or never*) to 5 (*every day of the week*)
Life Satisfaction (5) + Life Satisfaction-1 (1)	Satisfaction With Life Scale, German version (Janke and Glöckner-Rist, 2012 & Beierlein et al., 2015)		0.84	0.87	0.87	1 (*strongly disagree*) to 7 (*strongly agree*) + 1 (*not satisfied*) to 100 (*completely satisfied*)
Study Satisfaction (2)	Kurzfragebogen zur Erfassung der Studienzufriedenheit (FB-SZ-K; Westermann et al., 2018)					0 (*not true at all*) to 100 (*completely true*)
Academic Performance (2)	Original items					1 (*almost never*) to 5 (*almost always*)
Positive and Negative Affect (20)	Positive and Negative Affect Schedule, German version (Breyer and Bluemke, 2016)	PA	0.88	0.9	0.92	1 (*not at all*) to 5 (*extremely*)
		NA	0.88	0.85	0.89	
Self-Efficacy (3)	Allgemeine Selbstwirksamkeit Kurzskala (Beierlein et al., 2014)		0.82	0.83	0.85	1 (*not true at all*) to 5 (*completely true*)
Locus of Control (4)	Internale-Externale-Kontrollüberzeugung-4 (Kovaleva et al., 2012)	IC	0.67	0.62	0.71	1 (*not true at all*) to 5 (*completely true*)
		EC	0.48	0.49	0.62	
Coping (12)^1^	Brief Cope, German version (Knoll et al., 2005)	Prob	0.72	0.73–0.82	0.49–0.68	1 (*not at all*) to 4 (*very much*)
		Emo	0.680	0.68–0.74	0.65–0.75	
		Avo	0.591	0.63–0.76	0.52–0.72	
Perceived Stress (10)	Perceived Stress Scale 10, German version (Schneider et al., 2020)		0.88	0.86	0.85	0 (*never*) to 4 (*very often*)
Depression (8)	PHQ-8^a^, a section of the Health Questionnaire for Patients (Löwe and Spitzer, 2002)		0.81	0.85	0.8	0 (*not at all*) to 3 (*almost every day*)
States^b^			T0	T1	T2	
Character Strengths (24)	Character Strengths Rating Form (Gander et al., 2022)		0.90–0.94	0.92–0.95	0.92–0.95	0 (*not at all*) to 7 (*all the time*)
Mindfulness (12)	Multidimensional State Mindfulness Questionnaire (Blanke and Brose, 2017)		0.73–0.78	0.74-0.82	0.77–0.81	0 (*does not apply at all*) to 6 (*applies strongly*)
Gratitude (3)	Daily Gratitude Scale (Oosterhoff et al., 2022)		0.72–0.85	0.78-0.86	0.78–0.86	1 (*not at all*) to 7 (*very often*)
Self-Compassion (6)	State Self-Compassion Scale Short-Form, German translation (adapted from Hupfeld and Ruffieux, 2011)		0.68–0.71	0.70-0.74	0.69–0.76	0 (*does not apply at all*) to 6 (*applies strongly*)
Affect (6)	EAE (Blanke and Brose, 2017)		0.82–0.87	0.83–0.87	0.77–0.88	1 (*not at all*) to 5 (*very much*)
Coping (12)^1^	Brief Cope, German version (Knoll et al., 2005)					1 (*not at all*) to 4 (*very much*)
Perceived Stress (4)	Perceived Stress Scale Short-Form (Cohen and Williamson, 1988)		0.73–0.79	0.71–0.81	0.57–0.76	0 (*never*) to 4 (*very often*)
Procrastination (2)^1^	Academic Procrastination State Inventory, German version (Patzelt and Opitz, 2014)	Prob	0.69–0.82	0.69–0.8	0.62–0.81	1 (*never*) to 5 (*always*)
		Emo	0.67–0.74	0.61–0.86	0.7–0.83	
		Avo	0.61–0.8	0.55–0.79	0.59–0.76	
Relaxation (2)^1^	Relaxation State Questionnaire (Steghaus and Poth, 2022)					0 (*does not apply at all*) to 100 (*applies strongly*)
Physical Health (1)^1^	Short-Form-Health-Survey (Drixler et al., 2020)					0 (*very bad*) to 100 (*very good*)
Sleep Quality (1)	Sleep Quality Scale (Snyder et al., 2018)					0 (*terrible*) to 10 (*excellent*)

Assessment times: T0 – baseline, T1 – after the training, T3–6 month follow up. PA = Positive Affect; NA = Negative Affect. IC = Internal (Locus of) Control; EC = External Control. Prob = Problem-Focused Coping; Emo = Emotion-focused Coping, Avo = Avoidance-based Coping; H = Humor; PR = Positive Reframing.

a The ninth item, which asks about suicidal thoughts, was removed on the recommendation of the ethics committee, we refer to the remaining eight items as PHQ-8.

b State assessment: T0, T1, and T3 each consisted of 4 measurement time-points.

1 For economic reasons the scales were shortened.

### 2.5 Data analysis

#### 2.5.1 Power analysis

We conducted an *a priori* power analysis using G*Power to determine the required sample size for the analysis of variance (Faul et al., 2007). With an *α* = 0.05, a power of *β* = 0.80, to detect a mean effect of *g* = 0.34 (based on a meta-analysis on the effectiveness of multicomponent PPI, here for wellbeing as an outcome; Hendriks et al., 2020) and an assumed correlation of the measurement times of *r* = 0.5, a minimum of 58 subjects is required to effectively determine the interaction of group and time in a repeated measures ANOVA.

#### 2.5.2 Missing data

The analysis revealed a high proportion of missing values at the post-training time points, mainly due to participant dropout. To counteract potential systematic bias in the study sample, we analyzed according to the intention to treat principle (Twisk et al., 2020). We addressed the missing values using multiple imputations (cf. Twisk et al., 2020). The predictors used were previous measurements of the variable to be imputed (e.g., mindfulness at t1 was predicted by mindfulness at t0). We ran 20 imputations. For trait level constructs, estimating missing values was possible for most participants. However, for trait level constructs, it was often only possible to estimate values for parts of the sample, resulting in fewer analyzed cases.

#### 2.5.3 Statistical analysis

Mixed ANOVA were used to analyze trait measures. The factors were experimental group (intervention group vs. control group) and time (t0, t1, t2). Bonferroni-corrected pairwise comparisons were used to assess within- and between-group differences.

To analyze state measures, multilevel models were used. Fixed effects were calculated for group, time, and the group by time interaction. Additionally, a random slope was calculated for time. The group variable was coded as 0 = intervention group and 1 = control group. For each time point, the four daily state assessments were combined, resulting in three levels for the time variable (pre, post, and follow-up, respectively). While combining the assessments in this way obscures day to day variance, it is vital to assure that potential pre- to post-intervention changes become noticeable and are not suppressed. Because non-linear effects over time were expected, three models were calculated for each outcome variable, one per comparison of measurement points (i.e., t0–t1, t0–t2, t1–t2). A mediation analysis of trait level changes of primary on secondary outcomes was run, and results are reported in the OpenScienceFramework. We used the software IBM SPSS Statistics 25 (IBM SPSS Statistics (RRID: SCR_016479)).

## 3 Results

At baseline, the intervention group showed higher scores in problem-focused coping, avoidance-based coping, and wellness behavior. The intervention group also had more participants in higher semesters (see Table 2).

**Table 2:** Group differences at baseline.

Variable	Sub-categories	Intervention group *n* or mean (% or *SD*)	Control group	*p*
		*n* = 131	*n* = 84	
Gender	Female	107 (49.77%)	63 (29.3%)	0.79
	Male	19 (8.84%)	16 (7.44%)	
	Diverse	3 (1.4%)	2 (0.93%)	
	Not declared	1 (0.47%)	1 (0.47%)	
	Missing	1 (0.47%)	2 (0.93%)	
Semester	1	33 (15.35%)	32 (14.88%)	<0.001*
	2	18 (8.37%)	1 (0.47%)	
	3	33 (15.35%)	34 (15.81%)	
	4	8 (3.72%)	0	
	5	11 (5.12%)	9 (4.19%)	
	6	4 (1.86%)	2 (0.93%)	
	7+	23 (10.7%)	4 (1.86%)	
Course of study	Bachelor	96 (44.65%)	72 (33.49%)	0.16
	Master	10 (4.65%)	3 (1.4%)	
	Diploma	1 (0.47%)	1 (0.47%)	
	State Examination	14 (6.51%)	2 (0.93%)	
	Other	9 (4.19%)	4 (1.86%)	
Age		23.03 (4.27)	22.78 (4.82)	0.69
Depression		8.36 (4.18)	8.27 (4.9)	0.88
Stress		19.44 (6.38)	18.66 (6.25)	0.37
Character strengths		18.73 (2.95)	19.44 (2.95)	0.09
Mindfulness		2.47 (0.48)	2.54 (0.49)	0.29
Positive reframing		4.24 (1.09)	4.5 (1.14)	0.10
Self-compassion		2.82 (0.67)	2.87 (0.65)	0.55
Humor		19.65 (4.87)	20.7 (5.49)	0.14
Positive affect		30.55 (6.68)	30.3 (7.07)	0.79
Negative affect		21.99 (7.47)	20.48 (7.4)	0.15
Problem-focused coping		2.8 (0.63)	2.6 (0.61)	0.02*
Emotion-focused coping		2.43 (0.67)	2.39 (0.63)	0.65
Avoidance-based coping		1.54 (0.53)	1.49 (0.47)	0.46*
Self-efficacy		3.41 (0.71)	3.6 (0.74)	0.07
Internal locus of control		3.86 (0.69)	3.99 (0.7)	0.16
External locus of control		2.44 (0.73)	2.34 (0.74)	0.37
Wellbeing		51.75 (18.44)	52.05 (20.15)	0.91
Wellness behavior		3.46 (0.59)	3.24 (0.54)	0.005*
Satisfaction with life		23.27 (5.45)	24.09 (5.52)	0.29
Satisfaction with studies		73.69 (20.32)	75.52 (19.53)	0.52
Study performance		3.49 (0.96)	3.54 (0.97)	0.70

For categorical variables, the numbers of observations are presented, and the *p*-value refers to a chi-square contingency test. For metric variables, the mean is presented, and the *p*-value refers to an independent samples t-test. Bold values are significant at the *p* = 0.05 level.

### 3.1 Trait measures

Tables 3, 4 show the results for the analysis of trait measures (primary and secondary outcomes, respectively). The time × group interaction is significant for the constructs mindfulness, positive reframing, and self-compassion (*p*s<0.05). Therefore, the groups’ scores on these constructs changed differently over time. Pairwise comparisons revealed significant improvements within the intervention group for mindfulness, positive reframing, and self-compassion (all *p*s<0.05). Further, other than self-compassion, there are no variables showing a significant difference favoring the intervention group over the control group post-intervention.

**Table 3:** Mixed ANOVA results for primary outcome measures (imputed data).

Effect	Time	Group	Time × Group
Character strengths	*F*(1.961, 403.853) = 2.02	*F*(1, 206) = 0.87	*F*(1.961, 403.853) = 1.49
Mindfulness	*F*(1.946, 400.95) = 1.94	*F*(1, 206) = 0.3	*F*(1.946, 400.95) = 4.76*
Positive reframing	*F*(1.969, 405.548) = 5.29*	*F*(1, 206) = 0.29	*F*(1.969, 405.548) = 3.94*
Self-compassion	*F*(1.819, 372.933) = 17.60*	*F*(1, 205) = 0.68	*F*(1.969, 372.933) = 5.86*
Humor	*F*(1.892, 387.954) = 2.64	*F*(1, 205) = 0.18	*F*(1.892, 387.954) = 2.99

Values marked with an asterisk (*) are significant at the *p* = 0.05 level. Bold values are significant at the *p* = 0.05 level.

**Table 4:** Mixed ANOVA results for secondary trait measures (imputed data).

Effect	Time	Group	Time × Group
Depression	*F*(1.98, 393.44) = 2.38	*F*(1, 209) = 0.19	*F*(1.98, 393.44) = 0.38
Stress	*F*(1.97, 412.21) = 1.21	*F*(1,209) = 0.16	*F*(1.97, 412.21) = 2.62
Positive affect	*F*(1.92, 396.91) = 1.15	*F*(1,207) = 0.22	*F*(1.92, 396.91) = 0.776
Negative affect	*F*(1.96, 405.07) = 5.51*	*F*(1,207) = 0.87	*F*(1.96, 405.07) = 1.23
Problem-focused coping	*F*(1.98, 409.52) = 1.20	*F*(1, 207) = 3.95*	*F*(1.98, 409.52) = 1.68
Emotion-focused coping	*F*(1.98, 409.26) = 0.51	*F*(1, 207) = 0.53	*F*(1.98, 409.26) = 0.94
Avoidance-based coping	*F*(1.96, 406.61) = 4.14*	*F*(1, 207) = 0.99	*F*(1.964, 406.61) = 0.7
Wellbeing	*F*(1.93, 404.04) = 1.06	*F*(1, 209) = 0.30	*F*(1.93, 404.04) = 0.41
Satisfaction with life	*F*(1.90, 396.29) = 5.03*	*F*(1, 209) = 1.83	*F*(1.9, 396.29) = 0.7
Internal locus of control	*F*(1.97, 406.31) = 4.54*	*F*(1, 206) = 0.19	*F*(1.97, 406.31) = 2.59
External locus of control	*F*(1.97, 405.89) = 2.83	*F*(1, 206) = 0.14	*F*(1.97, 405.89) = 1.34
Study satisfaction	*F*(1.91, 392.28) = 20.95*	*F*(1, 205) = 0.66	*F*(1.91, 392.28) = 0.53
Study performance	*F*(1.93, 394.8) = 1.7	*F*(1, 205) = 0.19	*F*(1.93, 394.8) = 0.53
SL-1	*F*(1.99, 160.98) = 4.1*	*F*(1, 81) = 0.87	*F*(1.99, 160.98) = 1.28
Wellness behavior	*F*(1.93, 403.3) = 1.71	*F*(1, 209) = 5.6*	*F*(1.93, 403.3) = 2.5
Self-efficacy	*F*(1.98, 407.37) = 7.45*	*F*(1, 206) = 2.46	*F*(1.98, 407.37) = 0.87

Only non-imputed data available for SL-1. Values marked with an asterisk (*) are significant at the *p* = 0.05 level. Bold values are significant at the *p* = 0.05 level.

### 3.2 State measures

From t0 to t1, significant improvements within the intervention group were observed for self-compassion, positive affect, relaxation, subjective physical health, and subjective sleep quality compared to passive control participants (see Table 5). However, significant increases in negative affect were also observed in the intervention group. At t2, the improvements in self-compassion and positive affect remain in the intervention group (see OpenScienceFramework for comprehensive results).

**Table 5:** Multilevel model parameters for state measures between T0 and T1 (imputed).

Variable	Time	Group	Group × Time	Random slope time
Character strengths	−0.01	−0.02	0.15	0.19*
Gratitude	−0.05	−0.08	0.23	0.51*
Mindfulness	−0.11	−0.05	0.15	0.10*
Self-compassion	−0.04	−0.07	0.26*	0.14*
Stress	0.48	0.21	−0.51	2.04*
Problem-focused coping	0.06	−0.04	0.03	0.11*
Emotion-focused coping	>−0.01	0.04	0.17	0.17*
Avoidance-based coping	0.09	0.04	−0.02	0.04*
Relaxation	−5.20	−3.13	13.36*	210.89*
Positive affect	−1.05*	−0.27	1.33*	2.65*
Negative affect	−0.44	−0.05	0.61*	1.16*
Procrastination	−0.19	0.13	0.01	0.51*
Sleep quality	−0.25	−0.07	0.58*	1.16*
Subjective physical health	−2.14	−2.77	7.06*	263.89*

Only non-imputed data available for relaxation, sleep quality, and subjective physical health. Values marked with an asterisk (*) are significant at the *p* = 0.05 level. Bold values are significant at the *p* = 0.05 level.

### 3.3 Mediation

No mediating effects of the module constructs (character strengths, mindfulness, positive reframing, self-compassion, and humor) were found for any of the health-related outcomes of interest (wellbeing, depression, stress, positive affect, negative affect, and satisfaction with life). The comprehensive results can be found in the OpenScienceFramework repository.

### 3.4 Intervention compliance

Descriptive statistics show that on average 42% of participants used the learned exercise(s) at home. Only an average 14% of those participants reported using the exercises every day or 5–6 days of the week following the workshop, whereas an average of 82% used the exercises one to four times during that week. On average, 48% of those participants rated the exercises as rather or very useful, 45% rated them a little or somewhat useful, and 0.6% rated them as not useful at all. Per workshop, an average of 83% of intervention group participants who completed the compliance questionnaires attended the workshop.

Grouping the intervention group into a group that used any of the exercises at home vs. participants who used none of the exercises at home (74 vs. 57 participants, respectively), shows a significant difference after the intervention for positive reframing (*t*(215) = 2.85, *p* = 0.005) and negative affect (*t*(213) = −3.005, *p* = 0.003). However, no significant differences were observed for any other analyzed outcomes (all *p*s > 0.05).

Following up on the difference between those groups pertaining to positive reframing, we have re-run the repeated-measures analysis across all three measurement points, grouping the intervention group by exercise use, we find that there is a significant between-group effect (*F*(1) = 5.839, *p* = 0.021) (original data, all imputation sets also show *p* < 0.05). There is also a significant main effect of time in all imputed data sets, but not in the original data (*F*(2) = 1.003, *p* = 0.372, all other *p*s < 0.05). There is no significant time by exercise use interaction term (all *p*s > 0.05). These results suggest that there is a time-independent difference in positive reframing between the people who did and the people who did not use any exercises at home.

Find the full results for the compliance analysis in the OpenScienceFramework repository.

## 4 Discussion

This quasi-experimental study aimed to evaluate a 6-week multicomponent PPI within a university student sample via trait and state level assessments before and after the intervention, as well as at 6-month follow-up. The intervention group showed significant post-intervention improvements in trait self-compassion, mindfulness, and positive reframing, whereas the control group did not. While self-compassion and mindfulness improvements persisted at the 6-month follow-up, the improvements in positive reframing were not maintained. These findings align with previous research demonstrating that interventions targeting specific domains such as mindfulness, self-compassion, or positive reframing effectively enhance those particular constructs among university students (Dryden et al., 2023; Haukaas et al., 2018; de Sousa et al., 2021). At the state level, we found improvements in self-compassion, positive and negative affect, relaxation, subjective physical health, and sleep quality, immediately post-training.

Evidence shows that even brief interventions, such as 90 min of mindfulness training, can effectively increase positive psychological constructs like mindfulness among students, suggesting a singular effect of individual modules (de Sousa et al., 2021). Since we evaluated the impact of the 6-week intervention overall without weekly assessments of effectiveness, it is not clear which modules were driving the observed changes. However, existing evidence suggests that mindfulness, self-compassion, and positive reframing are interrelated, indicating potential synergistic effects within our intervention (Neff and Germer, 2013; Pogrebtsova et al., 2018; Shapiro et al., 2007). Furthermore, our PPI enhanced different states, including self-compassion, positive/negative affect, relaxation, physical health, and sleep quality, immediately post-training. Notably, changes in self-compassion and positive affect were sustained over time. These findings align with previous research showing state enhancement after PPIs (Breines and Chen, 2013; Congard et al., 2020). However, most studies examining state changes in PPIs have primarily focused on single-component interventions, particularly mindfulness interventions (Breines and Chen, 2013; Kiken et al., 2015; Mahmood et al., 2016). Since negative affect also increased initially, together with a sustained increase in self-compassion, this could be a backdraft effect of PPIs (e.g., in self-compassion), where a confrontation with negative emotions or experiences induces negative affect, but self-compassion can help in coping with it (Ewert et al., 2021). While this effect was not hypothesized or conceptually embedded in our study, it is a plausible explanation based on recent literature (Hoffmann et al., 2024). Thus, our study demonstrates promising results, highlighting the necessity for further research to explore state changes following multicomponent PPIs.

Contrary to our expectations, no significant changes occurred in other psychological outcomes on trait- or state-levels (such as character strengths and coping humor). Similarly, most health-related outcomes, including depression and wellbeing, did not improve following the intervention, in contrast to previous research findings (Chilver and Gatt, 2022; Hendriks et al., 2020; Hobbs et al., 2022). Interestingly, our results suggest that state–trait relationships may vary across psychological constructs. For example, both state- and trait changes occurred in self-compassion, whereas for mindfulness, only the trait level changed. According to Whole Trait Theory (Schmitt and Blum, 2020), the development of traits is thought to result from accumulated state-level experiences. However, our results imply that trait shifts can occur even in the absence of observable state-level changes—or that our state-level measurement was not sensitive enough to detect micro-level fluctuations. These results underscore the importance of assessing both levels distinctly, as well as the need for finer-grained, temporally sensitive measures to better understand how daily experiences translate into enduring personal change. Several factors could explain the absence of state-level effects. The intervention’s duration may have been insufficient, as interventions lasting 9–12 weeks have shown particular effectiveness in increasing psychological wellbeing (Hendriks et al., 2020). Furthermore, changes in module outcomes could be temporary and not fully captured by our pre- and post-training assessments. The daily diary schedule was a low-threshold implementation of daily data, but a more frequent assessment (e.g., multiple times per day) might illustrate more fluctuation in daily experiences and, thus, micro-level effects of the intervention (Wrzus and Neubauer, 2023). Therefore, it remains unclear whether the modules (e.g., targeting humor and character strengths) were ineffective or whether the measurement was inadequate to detect changes. We recommend more fine-grained assessment (e.g., multiple ambulatory assessments, weekly evaluation following each module). Lastly, not being able to estimate data for all missing values may have led to reduced statistical power, making our tests not sensitive enough to detect changes.

Moreover, participants may not have fully integrated the exercises into their daily lives, which may have weakened the outcomes, especially in the long term. For example, consistent effort and sustained practice of PPI exercises are crucial for reducing depressive symptoms (Seligman et al., 2005). Although each module contained recommendations, exercises, and motivational incentives for daily practice, participants used them to varying degrees. Future PPIs should encourage participants to engage more actively in the exercises at home and evaluate the implementation more thoroughly.

Finally, we must acknowledge the context of the implementation. Data collection started during the COVID-19 pandemic, with depressive symptoms, anxiety, suicidal ideation, feelings of loneliness, and stress being particularly high among university students (Aristovnik et al., 2020; Kohls et al., 2023; Tsiouris et al., 2023). On the one hand, this might increase their willingness to participate in the intervention to address these issues. On the other hand, since the PPI was not a therapeutic intervention, students might have had different expectations or sought more individualized, professional support that go beyond universal, group-based PPIs.

### 4.1 Strengths, limitations, and outlook

The study examined the impact of a 6-week, multicomponent PPI on state and trait levels of positive psychological skills and relevant health-related outcomes in German university students. It provides evidence for the feasibility of the intervention and its impact on self-compassion, positive reframing, and mindfulness over time. The peer education approach also allows students of relevant fields (e.g., psychology) to gather work experience and broaden their skill set. Nevertheless, the study has some limitations. Most notably, the study lacks randomization due to it being implemented as a university mental health initiative. Therefore, intervention participants were expecting to enroll in a program that supports their mental health. This expectation may already create a beneficial effect for them, and lead to higher motivation (Constantino et al., 2011). Furthermore, the intervention group is larger than the control group and differs on several baseline measures (e.g., problem-focused coping, or wellness-behavior), potentially introducing bias into their relationships on other variables. Future studies should employ randomized allocation, ideally with individualized module assessments (e.g., dismantling studies) to disentangle specific effects and with different types of controls (e.g., active controls, online vs. face-to-face groups). This way, other factors like group support, positive expectations, or trainer commitment can also be examined (Goleman and Davidson, 2017). It further allows for an analysis of the active components that make up the intervention effect, informing future interventions which modules are more effective than others (although one must beware of survivorship bias and not falsely reinforce modules that already work while neglecting the modules that need reinforcement). Secondly, the sample was homogeneous, predominantly young, highly educated, White, and female, thus limiting the findings’ generalizability (Oyserman et al., 2002). Future studies with more diverse samples, including participants from different cultural backgrounds and older individuals, are necessary for broader applicability. Furthermore, comparing clinical (e.g., students with depressive symptoms), subclinical (e.g., students with high levels of neuroticism), and non-clinical student samples would offer valuable insights into potential differential effects, revealing practical implications for each group. Thirdly, to reduce participants’ burden in our ambulatory assessment of daily state-like variables, we reduced the number of items per construct. For example, our sleep quality measurement consisted of only one item. While this approach is face valid, longer measures would be preferable in future research to picture a broader measure of several constructs. Additionally, the internal consistency of some measures was below adequate levels (e.g., Locus of Control, Brief-COPE). Fourthly, the absence of randomization may introduce self-selection bias, indicating that participants in the intervention group may have been more motivated and optimistic. Another limitation concerns missing values and participant dropout. While multiple imputations are the current preferred method for handling missing data (Allison, 2010), they are estimations that may significantly differ from actual values (Baltes-Götz, 2013). Furthermore, estimating missing state values has its limitations; for most variables only a few missing values could be imputed and for others none (e.g., sleep quality, subjective physical health). Especially for state-level measures it was often not possible to estimate values for all participants, reducing the statistical power for state-level analyses Therefore, results for, e.g., relaxation, subjective physical health, and subjective sleep quality may be biased by dropout. To address the challenge posed by the high dropout rate, future research should consider employing other strategies aimed at reducing participant-dropout (Scharfstein et al., 2012), most importantly by offering higher incentives for fully completing all questionnaires.

Given that the study exclusively utilized self-report measures, potential biases, such as social desirability (Fisher and Katz, 2000), may have influenced the results. Employing more objective measures, including behavioral assessments or evaluations by third parties (e.g., fellow students, parents, siblings, or partners), might mitigate such biases. A more fine-grained ambulatory assessment (e.g., multiple times a day across multiple days) could enhance ecological validity, reduce potential memory effects, and allow for an investigation of knowledge translation in everyday life (Shiffman et al., 2008; Wrzus and Neubauer, 2023).

A potential confounding factor is that control group participants could have had contact with intervention group participants, during which they may have exchanged information about the intervention’s contents. Thereby the control group would have been exposed to parts of the intervention, potentially affecting their outcomes.

Pertaining to practical implications, peer-to-peer multi-component positive psychological interventions are feasible and draw much interest from students. Participants generally comply with the exercises and consider them rather useful, although the effect of using the exercises at home remains unclear. Organizationally, such an intervention can be employed through the university’s sports and wellbeing administration. Intervention facilitators can be students with little to some knowledge in psychology with a training period of 2–3 sessions rehearsing the workshop and getting acquainted with the basic information on their presented module. To this end, a program supervisor with extended knowledge in positive psychology is needed. Further, some sort of incentive for the facilitators would be sensible, e.g., course credit, or a monetary reimbursement. Future studies could include staff members (e.g., teachers) into the intervention, drawing on findings that students may benefit from their teachers’ wellbeing as it creates a learning atmosphere that fosters wellbeing (Lo and Punzalan, 2025). This is promising for whole campus initiatives that aim to foster educator and student wellbeing concurrently, thus creating shared experiences and potentially, a positive social identity.

### 4.2 Conclusion

The current high levels of stress and mental health impairment among students attributed to the aftermath of the pandemic, multiple crises, and uncertainty about the future (Kohls et al., 2023; Zimmer et al., 2021) underscore the necessity for PPIs, which could boost students’ mental health and wellbeing. Since robustly evaluated interventions to enhance students’ mental health in Germany remain rare (Schweighart et al., 2023), this study provides a significant contribution. The findings of this research support the hypothesis that PPIs facilitate the development of positive psychological resources such as self-compassion, mindfulness, and positive reframing among university students.

## Data Availability

The datasets presented in this study can be found in online repositories. The names of the repository/repositories and accession number(s) can be found at: https://osf.io/mn4yz/?view_only=17c795c9507a4fb9a53bd052a2e1b037.

## Footnotes

^1^ https://www.soscisurvey.de
